# Current perspectives of cardio‐oncology: Epidemiology, adverse effects, pre‐treatment screening and prevention strategies

**DOI:** 10.1002/cam4.5980

**Published:** 2023-04-27

**Authors:** Jakub Bychowski, Wojciech Sobiczewski

**Affiliations:** ^1^ 1st Department of Cardiology Medical University of Gdansk Gdansk Poland

**Keywords:** cancer treatment, cardio‐oncology, cardiotoxicity, cardiovascular disease

## Abstract

Cancer and cardiac diseases are the most prevalent causes of death in most developed countries. Due to the earlier detection and higher effectiveness of treatment, more patients survive the disease and have a long life expectancy. As the post‐cancer population is growing, an increasing number of patients will be diagnosed with sequelae of those therapies, most often affecting the cardiovascular system. Although the risk of cancer recurrence decreases within years, the risk of cardiac complications—for example left ventricle (LV) systolic and diastolic dysfunction, arterial hypertension, arrhythmias, pericardial effusion and premature coronary artery disease remains elevated for decades after the completion of the therapy. The most common anticancer therapies that can cause adverse cardiovascular effects include chemotherapy—in particular anthracyclines, human epidermal growth receptor 2 targeted drugs and radiation therapy. A new field of research, cardio‐oncology, addresses this increasing risk, screening, diagnosis and prevention. This review aims to present the most relevant reports regarding the adverse cardiac effects of oncological therapy, including the most prevalent types of cardiotoxicity, methods of pre‐treatment screening and indications for prevention therapy.

## 1 INTRODUCTION

**FIGURE 1:**
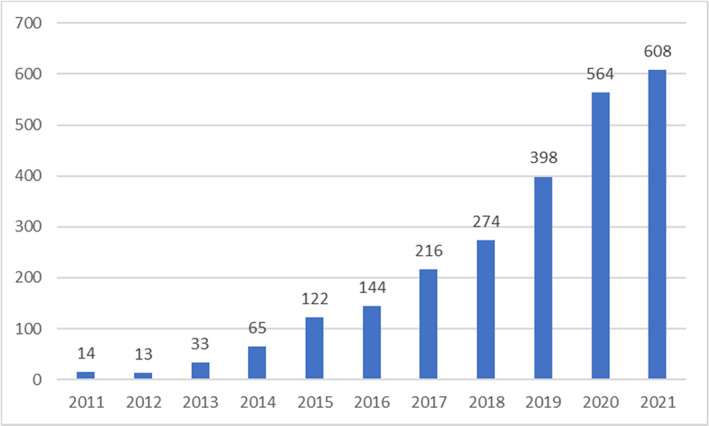
Articles considering cardio‐oncology in PubMed Research.

The cancer mortality rate has been declining over the past several decades due to the early detection, improved diagnosis and implementation of evidence‐based practices.^1^ In a GLOBOCAN study performed to estimate the worldwide incidence and mortality of the most prevalent cancers, the estimated mortality rate from lung cancer decreased from 36.8% in men in 2012 to 18% in 2020. In similar time, the mortality rate from breast cancer in women was reduced from 14.9% to 6.9%.^2^, ^3^ In 2016, the Polish Central Statistical Office reported 160,000 new cases of cancer in Poland.^4^ More than half of this group will pass through oncological therapy.^5^ Alongside this therapeutic success, there is an increasing population of cancer survivors at a higher risk of cardiovascular diseases (CVDs). Although the leading cause of death among this population is a recurrence of cancer or the primary disease progression, the second cause remains CVDs.^6^ Moreover, the risk of CVDs in this group remains elevated for up to 45 years after therapy. In contrast, the risk of cancer recurrence decreases after 5 years in the majority of cancers.^7^ Increasing interest in cardio‐oncology exists, which is presented in Figure 1 as the progressive growth in the number of studies (including: clinical trials, retrospective analysis, review, systematic review) published in PubMed. The cardiovascular risk includes a wide range of CVDs. Conventional chemotherapy and some targeted therapies can lead to heart failure (HF), arterial hypertension (AH), radiation‐induced heart disease (RIHD) and rhythm disturbances. RIHD may result in pericardial effusion, cardiomyopathy, myocardial fibrosis and coronary artery disease (CAD).^5^, ^7^ Some of them retreat spontaneously; others are hardly irreversible.^5^ According to the type of treatment, these effects can be dose‐dependent, cumulative or progressive, even when the therapy declines.^5^ Additionally, prior to the therapy, cancer patients are typically older and have more cardiovascular risk factors and comorbidities, which is related to the increasing hazard of cardiotoxic effects^8^ These facts were not taken into consideration in many previous studies.

This review aims (1) to characterise the most frequent cardiovascular complications of oncological therapy and (2) to present the most relevant reports regarding pre‐treatment screening and the prevention of cardiotoxicity.

## 2 EPIDEMIOLOGY

Following information from the Central Statistical Office in Poland, CVDs remain the primary causes of death in the first semester of 2020.^4^ Due to the improved effectiveness of oncological therapy, the 5‐year survival rates of the most prevalent cancers are increasing. The 5‐year relative survival rate for breast cancer increased from 79% for patients diagnosed between 1984 and 1986 to 91% for patients diagnosed between 2008 and 2014, while the 1‐year relative survival rate for lung cancer rose from 34% for patients diagnosed between 1975 and 1977 to 47% for those diagnosed between 2011 and 2014. Moreover, it is estimated that more than 3.8 million American women have a history of breast cancer; 268,600 were diagnosed in 2019. At the same time, 571,340 men and women living in the United States have a history of lung cancer, and 228,150 were diagnosed in 2019. It is estimated that 451,700 leukaemia survivors are living in the United States, of whom 61,780 were diagnosed in 2019.^1^ The therapy for acute lymphoblastic leukaemia is still chemotherapy.^9^ According to Armstrong et al., the current 5‐year relative survival rate is 89% from birth to 19 years of age. Even decades after the completion of cancer treatment, childhood cancer survivors have a 15‐fold increased rate of HF, a 10‐fold higher rate of CVDs and a 9‐fold higher rate of stroke. Moreover, this group has a higher incidence of cardiovascular risk factors in the future compared with their siblings.^10^

As the Centres for Disease Control and Prevention's National Centre reported, more than 16.9 million Americans with a history of cancer were alive on 1 January 2019. This population is expected to increase to 22.1 million by 2030.^1^

## 3 ADVERSE EFFECTS OF ONCOLOGICAL THERAPY

### 3.1 Cancer therapy‐related cardiac dysfunction

This term recommended by the European Society of Cardiology (ESC) in the new 2022 Guidelines on Cardio‐oncology includes cardiac injury, cardiomyopathy and HF.^11^ Both systolic and diastolic insufficiency remain the most frequent consequence of oncological therapy. It can be caused by several groups of agents, including anthracyclines (doxorubicin in particular), human epidermal growth factor 2 (HER‐2) agents (e.g. trastuzumab), angiogenesis inhibitors (e.g. sorafenib and sunitinib), proteasome inhibitors and microtubule inhibitors. Even though the mechanism, dose dependence and rapidity of progression may differ, the sequelae will lead to the same effect.^11^, ^12^

Anthracyclines are the oldest oncological drugs. After more than 60 years of clinical use, they still have many indications, including lymphoma, leukaemia, osteosarcoma and breast cancer.^13^, ^14^, ^15^, ^16^ The most commonly used drugs are doxorubicin, mitoxantrone and epirubicin.^8^ The anti‐neoplasm anthracycline mechanism combines four general effects: disrupting DNA and RNA synthesis by intercalating between base pairs, producing free radicals that disrupt the DNA, inhibiting topoisomerase II (Top2), and evicting histones.^8^ The authors hypothesise that the mechanism of toxicity is directly related to its anticancer properties, including both inhibition of Top2 and induction of reactive oxygen species (ROS) generation.^17^, ^18^ Two isoenzymes of Top2 exist in human cells: Top2 alpha is highly expressed in actively proliferating cells, while Top2 beta is expressed in quiescent cells such as cardiomyocytes.^17^ Zhang et al. showed that cells with a shortage of Top2 beta are protected from the toxicity of anthracyclines.^19^ The primary classification of anthracycline‐based toxicity include acute, early and late mechanism. The types of cardiotoxicity were divided by the criterium of time (acute – within 2 weeks of treatment, early – within 1 year after the completion of therapy, late – >1 year after the completion of therapy), dose dependence (early and late) and reversibility (acute – usually reversible, early and late – hardly reversible). However, the process of anthracycline toxicity is thought to be more continuous, starting from myocardial damage to clinically relevant heart failure.^18^ The factors raising the risk of anthracyclines' toxic effects are female sex, pre‐existing conditions (CAD, diabetes, AH, renal failure), extremes of age (paediatric or elderly populations), combined therapy with microtubule inhibitors (e.g. paclitaxel and docetaxel) and concomitant radiation therapy (RT) involving the heart.^5^, ^12^ The toxicity of anthracyclines is considered dose‐dependent, cumulative and progressive.^5^ Although every dose is reported to be harmful, in some doses, the risk of cardiotoxicity is acceptable.^8^ In a retrospective analysis, Swain et al. revealed that the majority of events occurred when the cumulative doses exceeded 500 mg/m^2^. The estimated cumulative percentage of patients with HF in that study was 5% at a cumulative dose of 400 mg/m^2^, rising to 16% at a dose of 500 mg/m^2^ and 48% at a dose of 700 mg/m^2^.^20^ Instead of systolic dysfunction administration of anthracyclines may also result in diastolic function worsening. As the authors claim, the diastolic dysfunction not always lead to decrease in left ventricle ejection fraction (LVEF); however, it always affect the mortality. In a retrospective study conducted by Al‐Jaroudi et al., the diastolic dysfunction was the independent predictor of death in patients with normal LVEF, resulting in 1.78‐fold increase in mortality.^21^

Even though the invention of targeted therapies provides new opportunities for treating patients, it does not resolve the issue of cardiotoxicity. Trastuzumab and other HER‐2 targeted agents revolutionised the therapy of HER‐2‐positive breast cancer. Before that, the 5‐year relative survival rate was low due to its aggressive growth and metastatic potential. Trastuzumab is a humanised monoclonal antibody approved by the Food and Drug Administration in 1998 as the first‐in‐class agent. By blocking the HER‐2/neu receptor, it stops the activation of intracellular growth factors. As a result, intracellular tyrosine kinases remain unphosphorylated, which results in a disruption of cell growth. The cardiac dysfunction caused by trastuzumab is the result of the disruption in signalling between the receptor HER‐2/ERBB2 and ligand neuregulin. In cardiomyocytes, this pathway is responsible for growth, repair and homeostasis.^8^ In most cases, it led to asymptomatic depletion in LVEF. Simultaneous therapy with anthracyclines, age > 50 years, obesity, primary CVD, LVEF <50% and preceding surgery are all factors that increase risk.^5^, ^11^, ^22^ In contrast to anthracyclines, cardiac deprivation caused by trastuzumab usually starts during therapy and is fully reversible in most cases.^11^ Kitani et al. investigated the molecular mechanism of cardiac dysfunction after trastuzumab administration in a stem cell‐derived cardiomyocyte model. The authors administered therapeutic doses of trastuzumab, which impaired the contractility and calcium handling of cells; however, they did not claim cardiomyocyte death or sarcomere disorganisation.^23^ The results of that study clarify the cause of left ventricular dysfunction (LVD) reversibility. Von Minckwitz et al. investigated the multi‐drug therapy combining pertuzumab with standard chemotherapy and 1‐year treatment with trastuzumab in the Aphinity Trial. After 3 years, pertuzumab significantly improved the invasive disease‐free survival rate without any differences in the occurrence of HF, cardiac death or LVD in both groups.^24^ These results show that improvement in oncological therapy can be achieved without influencing the toxicity profile. However, in another study, the authors revealed an increase in LVD risk in the simultaneous therapy of trastuzumab and paclitaxel.^25^

CVD has also been reported in patients treated with inhibitors of vascular endothelial growth factor (VEGF) and tyrosine kinase inhibitors (TKIs). TKIs inhibit numerous tyrosine kinases, including adenosine monophosphate‐activated protein kinase and platelet‐derived growth factor, which could explain their effects on the myocardium.^8^ The reversibility of CVDs remains unclear. In some reports, patients treated with sunitinib benefited from a temporary suspension of the therapy; however, in another study, the authors revealed the apoptosis of cardiomyocytes. Telli et al. investigated the population of patients treated with sunitinib and revealed that 15% of patients developed symptomatic CVD, some of them irreversibly.^26^ In another study, sunitinib was reported to cause LVD in 19% of cases.^27^

Another group related to LVD is proteasome inhibitors. They were approved for clinical use in multiple myeloma and mantle cell lymphoma. The role of the proteasome is to degrade abnormal proteins marked by ubiquitin. In cancer cells, this pathway is upregulated.^8^ The anticancer mechanism of this group includes inhibition of proteasome by binding to one of its subunits. The proposed mechanism of cardiotoxicity includes oxidative stress on cardiomyocytes proceeding to apoptosis. Pre‐existing CVD and administering anthracycline simultaneously remain risk factors for cardiac dysfunction.^8^ Waxman et al. conducted a meta‐analysis of 2594 patients who were administered with carfilzomib, which revealed the occurrence of adverse cardiovascular effects in 18.1% of the patients.^28^ It should be emphasised that the population with a diagnosis of multiple myeloma is older and is likelier to have pre‐existing CVD. In a study of 30,000 patients with the aforementioned diagnosis, nearly 2/3 had CVD at baseline.^29^

Although some of the drugs may cause LVD when used in a single therapy, other groups may predispose to this effect when combined. Pentassuglia et al. reported an increased risk of cardiotoxicity in the combination of paclitaxel and HER‐2 inhibitors.^25^

### 3.2 Hypertension

Many oncological patients have a diagnosis of a well‐controlled hypertension before therapy, which complicates the pre‐treatment evaluation. Groups of anticancer drugs that contribute to AH are VEGF inhibitors, TKIs, HER‐2 targeted agents, platinum‐based chemotherapy agents, proteasome inhibitors and anthracyclines.^5^

VEGF inhibitors and TKIs are relatively new groups of drugs, with the first representative (bevacizumab) introduced shortly after 2000. The most common indications are breast, thyroid, hepatocellular and renal cancer.^5^ Drugs affect cells through two mechanisms: inhibiting VEGF or the VEGF receptor tyrosine kinase. TKIs have multiple grab points, which make them effective anticancer agents; nevertheless, it makes the aetiology of AH extremely complicated. In patients treated with VEGF inhibitors, the elevation of severe vasoconstrictor endothelin‐1 occurs, which is dose‐dependent.^30^ Another change that contributes to AH is the reduction in nitric oxide (NO) concentration in the blood.^31^ It has been asserted that the reduction of NO is the consequence of the elevation of the oxidative stress units superoxide anion (O_2_^−^) and hydrogen peroxide (H_2_O_2_). It has been proven that VEGF inhibitor‐dependent hypertension does not have to be related to an increase in O_2_^−^ or H_2_O_2_ levels, and the elevation in oxidative stress does not have to contribute to the rise in blood pressure. These results suggest that elevated ROS seems to be the consequence rather than the reason for AH.^31^ Another mechanism of AH is the elevation of aldosterone levels. Risk factors for AH are pre‐existing HF, previous anthracycline therapy and previous AH diagnosis. Although AH is the most prevalent cardiotoxicity effect of this therapy, with an incidence rate of 19–47%, the frequency of its onset varies among different representatives of this group.^12^ Versmissen et al. revealed that the incidence of AH started at 4% (sorafenib and vandetanib) and rose to 84% (axitinib).^32^ The influence of VEGF inhibitors on blood pressure is dose‐dependent.^33^

HER‐2 targeted agents are another group of drugs that can contribute to AH. The patomechanism include an inhibition of the neuregulin/ERBB2 pathway, which affects not only the LV but also vasomotor tone and sympathetic output. Lennemann et al. revealed that patients with breast cancer who received trastuzumab or lapatinib treatment had increased blood pressure, heart rate and levels of norepinephrine.^34^

### 3.3 Rhythm disturbances and QTc prolongation

Rhythm disturbances can occur during or after oncological treatment, and they can be the result of the oncological therapy, existing comorbidities or other medicine taken by the patient. Anthracyclines, microtubule inhibitors, antimetabolites (e.g. 5‐fluorouracil and capecitabine), proteasome inhibitors, TKIs and alkylating agents can cause arrhythmias during the therapy. The possible arrythmias include a wide spectrum from asymptomatic bradycardia to atrial fibrillation (AF), ventricular tachycardia and sudden cardiac death. The most prevalent abnormality, corrected QT interval (QTc) prolongation, can be the result of abnormal electrolyte levels or cancer treatment. The oncological drugs most frequently related to it are doxorubicin and lapatinib (with a risk of 11%–14%).^11^ The risk factors related to QTc prolongation are pre‐existing QTc prolongation, higher age, female sex, electrolyte disturbances and the following comorbidities: renal failure, hepatic failure, HF and CAD.^11^

Microtubule inhibitors are a group of drugs that were first introduced in the 1960s and were expected to be effective in treating cancer types with dynamic growth. The main indications are ovary, breast, non‐small cell lung and prostate cancers.^8^, ^35^ The antineoplastic effect of this group includes the stabilisation of guanosine diphosphate‐bound in the microtubules, which leads to inhibiting cell division. The paclitaxel and docetaxel toxicity is the result of decreased calcium amplitude in cardiomyocytes during contraction, which decreases the contractility of cardiomyocytes. Another factor predisposing patients to rhythm disturbances is polyethoxylated castor oil—a substance added to paclitaxel that can contrive histamine release and allergic reaction. CVDs related to a higher risk of arrhythmias are CAD and HF.^35^ The majority of the available data was collected from small retrospective studies, case studies or animal models. In an observational study including 100 patients treated with therapeutic doses of paclitaxel, the following arrhythmias were reported during an 8‐year follow‐up period (presented here by the percentage of all reported arrhythmias): 26% sinus tachycardia, 13% non‐specific T‐wave changes, 6% myocardial infarction, 4% prolonged QT interval, 4% left bundle branch block, 3% right bundle branch block, 3% sinus bradycardia, 2% premature atrial contractions, 2% atrial flutter and 1% AF. At the beginning of the study, 80% of the study population had at least one cardiac risk factor.^36^

### 3.4 Adverse effects of radiation therapy

It has been reported that more than 50% of patients with a diagnosis of cancer undergo RT.^12^ It can be applied in cancers of the breast, lung, oesophagus, thyroid gland and prostate as well as in mediastinal lymphoma or tumours of the head and neck^37^ and is used as a part of combined therapy in the majority of cases. The efficiency of RT is the effect of DNA disruption, local inflammation and tissue fibrosis.^5^ After repeated episodes of ischemia caused by inflammation, microvascular endothelial cells of the pericardium start a process of fibrosis. Epicardial changes are most clinically relevant in coronary arteries, where RT leads to intravascular inflammation and a reduction in the amount of oxygen and nutrients transported to the myocardium, which results in cardiomyocyte necrosis and fibrosis.^8^ In addition, RT leads to a prothrombotic state, vasospasm and elevated recruitment of monocytes and macrophages to tunica intima with increased inflow of lipoproteins.^38^ These combined alterations result in the acceleration of the existing atherosclerosis process or the initiation of new atherosclerotic plaques.^11^ It should be emphasised that the incidence of risk factors elevates the cardiotoxicity hazard, including higher total radiation dose, combined therapy with anthracyclines, irradiation of the heart, younger age (<25 years), previous CVD and the occurrence of risk factors.^38^ The clinical manifestations of RT toxic effects include pericarditis, CAD, valvular heart disease (VHD), LVD, AH and rhythm disturbances. All of these effects can occur years after the completion of the therapy.^8^ CAD as a result of RT differs from the disease not related to oncological therapy. Vasculopathy usually manifests as severe, diffuse, long and concentric lesions in coronary angiography.^11^ In most cases, patients are asymptomatic because of reduced pain sensation. The risk of CAD is increased within 10 years after the completion of the therapy. The localization of the culprit lesion is related to the radiation area and is generally localised in the proximal left anterior descending (LAD) or right coronary artery.^11^ Women with left‐sided breast cancer remain in the group with the highest risk of post‐RT CAD, with the highest risk of occlusions in the distal part of the LAD and diagonal artery, with 70% of lesions localised in the LAD.^37^ Compared to women with right‐sided breast cancer, this population has a 2.5 greater risk of CAD.^39^ Moreover, mortality from any cardiac cause is higher in left‐sided breast cancer patients.^37^ AH, diabetes, smoking and pre‐existing circulatory or respiratory diseases remain the additive risk factors of CAD occurrence among these patients.^38^ VHD usually becomes symptomatic years after RT, with a mean time of >20 years.^37^ The most commonly reported VHDs are aortic stenosis and mitral regurgitation.^11^ The risk of VHD development has been correlated with increasing RT doses.^5^, ^37^ The risk of LVD after RT rises 5 years after the therapy and is the result of damage to microcirculation, which leads to myocardial fibrosis and diastolic dysfunction.^40^ The specific adverse effect of head and neck radiation is AH. The mechanism includes direct damage of carotid sinus receptors, fibrosis of the arterial wall and accelerated atherosclerosis.^40^ Rhythm disturbance as a result of RT toxicity is a rare complication. The damage to the conduction system can be primary (due to radiation) or secondary (due to myocardial fibrosis). The clinical manifestations vary and include bundle branch block, fascicular block, III^o^ heart block and AF. Indications for anticoagulation therapy in patients with AF are identical to those in the general population; however, CHA_2_DS_2_‐VASc and HAS‐BLED risk scores have not been validated in this population.^11^ It must be emphasised that the majority of available data concerning patients who underwent RT 10 or more years ago when 3‐D imaging techniques were not so widely available.^38^ These changes make it hard to evaluate the toxicity of RT when administered using contemporary methods.

## 4 PRE‐TREATMENT SCREENING METHODS

Pre‐treatment cardiac risk evaluation should include complex assessment; it should be implemented in all oncological patients who are planned to receive potentially cardiotoxic treatment.^11^ There is no existing risk score for the majority of the population; also, none of the existing has been validated in a prospective study. According to the 2022 ESC Guidelines on Cardio‐oncology, the Heart Failure Association‐International Cardio‐Oncology Society (HFA‐ICOS) risk assessment tool should be considered for the pre‐treatment screening.^11^ Risk factors in cardio‐oncology can be divided into four groups: existing myocardial disease, cardiac risk factors, risk factors related to lifestyle and previous cardiotoxic cancer therapy.^41^ After the identification of existing risk factors, a screening scheme should be planned. According to the 2016 ESC Position Paper on Cardio‐oncology, switching the imaging technique throughout the therapy is discouraged, tests with the best reproducibility are recommended and radiation‐free imaging modalities that provide additional data imaging are preferred.^12^

### 4.1 Imaging techniques

The recommended imaging modality in cardio‐oncology is transthoracic echocardiography (TTE), due to its widespread availability, lack of radiation, non‐invasive character and relatively low cost.^11^, ^12^ The crucial limitation of this method is the variability between the examiners. The most commonly used parameter to evaluate the global systolic function of the LV is the LVEF measured in 3‐D echocardiography. If 3‐D imaging is not feasible, 2‐D echocardiography (Simpson's method) is recommended.^11^ An identical imaging technique is recommended during the whole therapy. Referring to the 2022 ESC Guidelines on Cardio‐oncology, cardiotoxicity is a decrease in the LVEF >10 percentage points to a value below normal (< 50%) confirmed 2–3 weeks after initial detection^11^ However, LVEF is considered a defective parameter due to the delay in its deterioration. Moreover, LVEF might not be reduced in hypertrophic cardiomyopathy. Recently, global longitudinal strain (GLS) has been considered the most accurate diagnostic tool.^42^ GLS is a parameter projected to describe the deformation of the myocardium in the cardiac cycle. The normal value of GLS was estimated in a meta‐analysis to be between −15.9% and−22.1%.^43^ Referring to the 2022 ESC Guidelines on cardio‐oncology, cardiotoxicity is a decrease in GLS > 15% compared to the baseline.^11^ The variability of GLS was assessed as lower than that of LVEF, with higher precision in measuring GLS among non‐experienced examiners.^44^ In addition, LVEF between 40% and 45% has an ambiguous prediction of mortality, whereas GLS is strictly correlated with mortality. In a meta‐analysis of 5721 patients, GLS was confirmed to be a strong predictor of all‐cause mortality.^45^ However, some studies are not confirming the superiority of GLS when compared to LVEF. The recently published results of SUCCOUR Trial presented no significant disparity between the results in oncological patients monitored by GLS or LVEF.^46^ To assess the diastolic function authors in the previous studies applied widely utilised parameters in clinical practice: mitral valve peak E‐ and A‐wave velocities, tissue doppler mitral annular e’ velocity and average E/e’.^47^ None specific parameter for cardio‐oncological patients was recommended in the newest ESC Guidelines.

In the 2022 ESC Guidelines on cardio‐oncology, several monitoring strategies were implemented, defining the specific frequency of examinations depending on the type of treatment, cumulative dose of cardiotoxic drugs, duration of the therapy and baseline risk factors.^11^ The first examination should be performed before therapy initiation to evaluate baseline and pre‐existing risk factors. In addition, control visits should be considered in patients with a high cumulative dose of anthracycline or mediastinal radiotherapy. Negishi et al. proposed a strategy that consists of imaging before treatment to evaluate risk factors and assess the indications for primary cardioprotective therapy and follow‐up imaging during therapy for the early detection of subclinical changes in myocardial systolic function. After‐treatment, imaging is recommended beginning 6–12 months after completion of the therapy in asymptomatic patients.^41^ Following the expert consensus of the European Association of Cardiovascular Imaging (EACVI) and the American Society of Echocardiography (ASE), asymptomatic patients without risk factors should be examined 10 years after RT. After that, in cases with no cardiac abnormalities, the next screening should be performed every 5 years. In the case of high‐risk patients (anterior or left‐side chest irradiation), screening should be performed after 5 years. In this population, repeated stress testing every 5 years should be planned.^48^ According to the 2022 ESC Guidelines on cardio‐oncology, patients with high end‐of‐treatment risk (min.1 from following criteria: baseline high or very high risk based on HFA‐ICOS risk tool; cardiotoxic therapy with high risk of future CVDs, moderate or severe CVD diagnosed during the therapy, new abnormalities in cardiac function in imaging assessment) should be monitored for the first 12 months after the therapy. After that time individual schemes depending on the cardiac risk and implemented treatment should proceed.^11^

Magnetic resonance imaging (MRI) plays a growing role as a screening method in the oncological population. It provides a reproducible assessment of LV volumes, mass and function.^49^ According to the expert consensus between EACVI and ASE, MRI is highly valuable in the screening and diagnosis of the oncological population.^48^ The disadvantages of MRI are its higher cost and lower availability compared to TTE. Moreover, due to the long acquisition time and specific conditions of some patients (e.g. claustrophobia and breath‐hold), it cannot be administered in every case. Cardiac function can be gauged by either the LVEF measure or by measuring myocardial strain. Myocardial strain is the measurement of myocardial deformation during the cardiac cycle on a specific axis (longitudinal, circumferential or radial) in comparison with its primary length.^49^ Peng et al. assessed the reference values of myocardial strain and evaluated LV parameters as −22.4% ± 2.9%, −24.3% ± 3.1%, and 79% ± 19.4% for longitudinal, circumferential and radial strain.^50^

Instead of the aforementioned methods, there is another advanced diagnostic tool, positron emission tomography, which offers advanced sensitivity for cardiotoxicity detection, although because of limited availability and low‐cost effectiveness, it is not widely used.

### 4.2 Biochemical markers

Biochemical markers remain one of the most clinically relevant diagnostic methods for evaluating the risk of cardiotoxicity. Precision, low cost and common availability are the essential advantages. The time delay and differences in laboratory procedures persist the main limitations. The strategy after the elevated result of the test remains unknown, although it has been researched whether abnormalities may help to identify patients developing systolic dysfunction.^11^, ^12^

The connection between elevated cardiac troponin I (cTnI) and the increased onset of systolic dysfunction is well researched during anthracycline therapy. Moreover, the maintenance and higher level of troponins (> 50 ng/L) were correlated with a greater reduction in LVEF.^51^ It is predicted that a normal level of cTnI has a strong negative predictive value for patients treated with trastuzumab. The role of cTnI during immunotherapy has been explored, although cases of troponin‐negative immunotherapy‐mediated myocarditis have been reported.^52^

Brain natriuretic peptide (BNP) and N‐terminal pro‐B type natriuretic peptide (NT‐proBNP) are widely utilised in cardio‐oncology, from screening examination before therapy to the detection of acute cardiotoxicity <24 h after exposure to anthracycline.^51^ In addition, a correlation was found between the level of NT‐proBNP and the deterioration of LVEF, which means that the level of NT‐proBNP predicts the severity of myocardial damage.^51^ Baseline NT‐proBNP measurement is recommended in high and very high risk patients.^11^

Recently, a few other markers have been researched to investigate their roles in cardiotoxicity diagnosis. Myeloperoxidase has been found to predict adverse cardiac effects after treatment with trastuzumab.^53^ No association has been found between elevated GDF‐15 or Galactin‐3 levels and cardiotoxicity; these markers can be elevated as a result of malignancy.^51^ The next investigated markers are non‐coding RNAs. Wang et al.'s study revealed that an elevated level of microRNA‐208a can be a useful indicator of high risk in patients with HF and malignancy; however, its role in cardiotoxicity remains unclear.^54^

## 5 PREVENTION STRATEGIES

The method of protection depends on clinical variables, stage of the therapy and results of additional examinations. There are no existing guidelines that indicate the moment of treatment initiation or recommended drugs. Documents regarding cardio‐oncology are presented in Table 1.

**TABLE 1:** Position papers considering cardio‐oncology.

Authors	Document	Year of publication
European Society of Cardiology	Position paper on cancer treatment and cardiovascular toxicity	2016
European Association of Cardiovascular Imaging, American Society of Echocardiography	Expert consensus for multi‐modality imaging evaluation of cardiovascular complications of radiotherapy in adults	2013
European Society of Cardiology	Guidelines on cardio‐oncology	2022
American Heart Association	Cardio‐oncology: vascular and metabolic perspectives: a scientific statement	2019
American Heart Association	Recognition, prevention and management of arrhythmias and autonomic disorders in cardio‐oncology	2021
Heart Failure Association, European Association of Cardiovascular Imaging, European Society of Cardiology	Role of cardiovascular imaging in cancer patients receiving cardiotoxic therapies: a position statement	2020
European Society For Medical Oncology	Management of cardiac disease in cancer patients throughout oncological treatment: consensus recommendations	2020

Following the 2022 ESC Guidelines on cardio‐oncology, preventive strategies include optimising lifestyle, cessation of risk factors and treatment of comorbidities.^11^ Physicians should avoid polypharmacy, drugs that can cause QTc prolongation or electrolyte disturbances. Anthracyclines should be administered at the lowest therapeutic doses in continuous infusion by liposomal delivery. Cardioprotective therapy should be considered in patients with low risk and high future exposure to anthracyclines. Patients with symptomatic HF must be screened in a cardio‐oncologic centre.^11^ Beta‐blockers, angiotensin‐converting enzyme inhibitors (ACEI), dexrazoxane and statins are drugs expected to have a cardioprotective effect.

The cardioprotective effect of beta‐blockers is a reduction in ROS and myocardial calcium overload. In addition, carvedilol has antioxidant properties and prevents lipid peroxidation; thus, nebivolol has a vasodilating effect thanks to the increase in NO level.^5^, ^12^ Recently, Xu et al. performed a meta‐analysis in which 844 patients from 11 studies were enrolled. The results showed that LVEF after chemotherapy was higher in the group with beta‐blockers, although this effect was significant only in patients with medium accumulative doses of anthracyclines (250 mg/m^2^–400 mg/m^2^), when beta‐blocker administration was longer than 6 months.^55^ In a prospective, double‐blind, randomised trial of carvedilol effects in preventing chemotherapy‐induced cardiotoxicity, the authors intended to assess the effectiveness of carvedilol in an HER‐2‐negative breast cancer group treated with anthracycline, cyclophosphamide and taxane. The results showed no significant changes in LVEF (measured by Simpson's method through apical 4 and 2 chambers views) between the two groups at the 6‐month follow‐up; however, in the carvedilol group, TnI levels after therapy were significantly lower and there were fewer cases of diastolic dysfunction compared to the placebo group, which suggests a possible carvedilol protective effect on the myocardium.^56^

The protective mechanism of ACEI includes the inhibition of ventricular remodelling.^57^ Gupta et al. assessed the role of enalapril in a randomised, double‐blind, clinical trial in which measurements of LVEF (2‐D echocardiography) and cardiac biochemical markers (cTnI, proBNP and CK‐MB) were performed. After 6 months of treatment, significant differences were detected in the LVEF and mean levels of cTnI and proBNP. The results suggest that enalapril can limit or stop the early cardiotoxicity of anthracyclines and prevent the development of late effects.^58^

Dexrazoxane might have a protective effect in patients treated with high cumulative doses of anthracycline. In the myocardium, anthracyclines bind to ferric cation (Fe^3+^), which contributes to the release of ROS and the beginning of apoptosis.^6^ Dexrazoxane chelates iron, which results in a reduction of oxidative stress. However, there are concerns that the administration of dexrazoxane reduces anthracycline's anticancer activity and raises the risk of secondary malignancies. In Macedo et al.'s meta‐analysis to evaluate this aspect, 2177 female patients with metastatic or locally advanced breast cancer treated with anthracyclines and HER‐2 agents were enrolled. Dexrazoxane therapy resulted in a lower risk of HF and a lower incidence of cardiac events, regardless of previous exposure to anthracycline. No differences occurred in the response rate or overall survival rate between the groups, and progression‐free survival was higher in the group with dexrazoxane.^59^

Statins are known for their ability to reduce cholesterol and triglyceride levels as well as their anti‐inflammatory and pleiotropic effects. The effectiveness of statins in LVEF preservation was evaluated in Seicean et al.'s retrospective cohort study, which enrolled 201 women with breast cancer into two groups (statin administration and control). The incidence of HF and cancer‐related mortality were significantly lower in the statin group irrespectively of other risk factors.^60^ The results from the another study comparing the administration of statins in low and medium cardiovascular risk patients with the diagnosis of breast cancer (NCT03971019) are expected in 2024.

The post‐oncological adverse effect that requires specific treatment is AH. The 2018 ESC/European Society of Hypertension Guidelines should be implemented in this population. Although several non‐pharmacological interventions exist, pharmacological therapy is inevitable in most cases.^61^ There are no individual recommendations for the oncological population. ACEI, angiotensin receptor blockers and calcium channel blockers (CCB) are the first‐line therapies. Diuretics and second‐generation beta‐blockers remain the second option. AH is strictly correlated with VEGF inhibitors. An increase in blood pressure has been observed even in the first cycle of treatment. The only recommendation in this group is to avoid non‐dihydropyridine CCBs (i.e. verapamil and diltiazem) due to their inhibitory effect on cytochrome P450, the enzyme that takes part in VEGF inhibitor metabolism. The specific feature of post‐VEGF inhibitors AH is the reversibility of this disease after the end of therapy.^62^

The possible prevention of cardiac dysfunction after doxorubicin and trastuzumab therapy was investigated by Milano et al. The authors administered treatment in rats with simultaneous intravenous administration of cardiac‐resident mesenchymal progenitor cells (CPCs) containing nanosized exosomes. The authors revealed that the administration of CPCs prevented cardiac dysfunction via a reduction in ROS generation.^63^

## 6 CONCLUSION

The development of oncological therapy causes increases in survival rates in numerous malignancies. In addition, the average age of the patient diagnosed with cancer increased and patients have more comorbidities than ever. These two aspects contribute to a higher incidence of CVDs in this population. Cardio‐oncology developed in response to the increasing needs of these patients. In this specific field, close cooperation between oncologists and cardiologists is indispensable. A simple risk scale for toxicity probability estimation should be created and verified in a prospective study. The recent 2022 ESC Guidelines on Cardio‐oncology deliver several recommendations to standardise the clinical practice which are mostly based on expert opinions. There is a strong need to initiate further studies to improve the care of oncological survivors.

## AUTHOR CONTRIBUTIONS

**Jakub Bychowski:** Conceptualization (equal); data curation (lead); resources (lead); software (lead); writing – original draft (lead). **Wojciech Sobiczewski:** Conceptualization (equal); supervision (lead); writing – review and editing (lead).

## FUNDING INFORMATION

This research received no external funding.

## CONFLICT OF INTEREST STATEMENT

None declared.

## Data Availability

Data sharing is not applicable to this article as no new data were created or analyzed in this study.
